# Alcohol Consumption Is Associated with Poor Prognosis in Obese Patients with COVID-19: A Mendelian Randomization Study Using UK Biobank

**DOI:** 10.3390/nu13051592

**Published:** 2021-05-10

**Authors:** Xiude Fan, Zhengwen Liu, Kyle L. Poulsen, Xiaoqin Wu, Tatsunori Miyata, Srinivasan Dasarathy, Daniel M. Rotroff, Laura E. Nagy

**Affiliations:** 1 Department of Inflammation and Immunity, Cleveland Clinic, Cleveland, OH 44195, USA; fanxiudexjtu@163.com (X.F.); Kyle.L.Poulsen@uth.tmc.edu (K.L.P.); wux@ccf.org (X.W.); miyatat@ccf.org (T.M.); dasaras@ccf.org (S.D.); 2 Department of Infectious Diseases, First Affiliated Hospital of Xi’an Jiaotong University, Xi’an 710049, China; liuzhengwen113@xjtu.edu.cn; 3 Department of Gastroenterology and Hepatology, Cleveland Clinic, Cleveland, OH 44195, USA; 4 Department of Molecular Medicine, Case Western Reserve University, Cleveland, OH 44195, USA; 5 Department of Quantitative Health Sciences, Cleveland Clinic, Cleveland, OH 44195, USA; rotrofD@ccf.org

**Keywords:** alcohol consumption, COVID-19, susceptibility, mortality, mendelian randomization, UK biobank

## Abstract

Background: Acute and chronic alcohol abuse has adverse impacts on both the innate and adaptive immune response, which may result in reduced resistance to severe acute respiratory syndrome coronavirus-2 (SARS-CoV-2) infection and promote the progression of coronavirus disease 2019 (COVID-19). However, there are no large population-based data evaluating potential causal associations between alcohol consumption and COVID-19. Methods: We conducted a Mendelian randomization study using data from UK Biobank to explore the association between alcohol consumption and risk of SARS-CoV-2 infection and serious clinical outcomes in patients with COVID-19. A total of 12,937 participants aged 50–83 who tested for SARS-CoV-2 between 16 March to 27 July 2020 (12.1% tested positive) were included in the analysis. The exposure factor was alcohol consumption. Main outcomes were SARS-CoV-2 positivity and death in COVID-19 patients. We generated allele scores using three genetic variants (rs1229984 (Alcohol Dehydrogenase 1B, *ADH1B*), rs1260326 (Glucokinase Regulator, *GCKR*), and rs13107325 (Solute Carrier Family 39 Member 8, *SLC39A8*)) and applied the allele scores as the instrumental variables to assess the effect of alcohol consumption on outcomes. Analyses were conducted separately for white participants with and without obesity. Results: Of the 12,937 participants, 4496 were never or infrequent drinkers and 8441 were frequent drinkers. Both logistic regression and Mendelian randomization analyses found no evidence that alcohol consumption was associated with risk of SARS-CoV-2 infection in participants either with or without obesity (All *q* > 0.10). However, frequent drinking, especially heavy drinking (HR = 2.07, 95% CI 1.24–3.47; *q* = 0.054), was associated with higher risk of death in patients with obesity and COVID-19, but not in patients without obesity. Notably, the risk of death in frequent drinkers with obesity increased slightly with the average amount of alcohol consumed weekly (All *q* < 0.10). Conclusions: Our findings suggest that alcohol consumption has adverse effects on the progression of COVID-19 in white participants with obesity, but was not associated with susceptibility to SARS-CoV-2 infection.

## 1. Introduction

Coronavirus disease 2019 (COVID-19), caused by severe acute respiratory syndrome coronavirus-2 (SARS-CoV-2), is a highly contagious, fast-spreading, and life-threatening infectious disease [1]. So far, it has spread to almost 200 countries and regions, infecting millions of people [2]. In its most serious presentation, COVID-19 can progress rapidly into acute respiratory distress syndrome (ARDS), multi-organ failure, and even death [3,4]. Thus, identifying potential risk factors for COVID-19 would be a substantial benefit to the public health.

In the midst of the COVID-19 pandemic, off-premise sales of alcohol have increased, according to Nielsen data [5,6]. Acute and chronic alcohol abuse have adverse impacts on both the innate and the adaptive immune response [7,8,9], and alcohol consumption is associated with increased susceptibility to pneumonia, tuberculosis, respiratory syncytial virus (RSV) infection, and acute respiratory distress syndrome (ARDS) [8,10,11]. Chronic alcohol abuse also exacerbates severity of influenza A virus infection by inhibiting influenza-specific CD8 T cell responses [9]. SARS-COV-2 contains a positive-sense, single-stranded RNA (+ssRNA) [12]. Recent data from both murine models of ethanol exposure and peripheral blood mononuclear cells (PBMCs) from patients with alcohol-associated hepatitis (AH) indicate that signaling by viral ss/dsRNA is disrupted by alcohol [13,14,15], analogous to impact of alcohol on signaling via bacterial products, such as lipopolysaccharide [16]. Therefore, we hypothesized that alcohol consumption may result in reduced resistance to SARS-COV-2 infection and promote the progression of COVID-19.

During the current COVID-19 pandemic, many misconceptions about the protective effects of alcohol in preventing COVID-19 have appeared in social media [17,18]. Although the World Health Organization (WHO) and other public health authorities have stressed that alcohol consumption does not destroy SARS-CoV-2 and may actually promote infection and accelerate disease progression because of the immunosuppressive effects of alcohol [19], many people around the world still believe that drinking alcohol helps prevent COVID-19 [17,20].

To date, there are no large population-based data evaluating the potential causal associations between alcohol consumption and COVID-19. Although randomized controlled studies (RCTs) are the ‘gold standard’ for studying the causal role of exposure factors in the development of diseases, it is not acceptable to expose subjects to alcohol for the purpose of causal inquiry. As an alternative, the non-interventional Mendelian randomization approach is increasingly used in clinical research [21,22]. Briefly, the Mendelian randomization approach is comprised of two main parts, first randomizing participants based on genetic variation, and then evaluating the causal relationship between exposure factors and disease. Genetic variants are used as ‘instrumental variables’ that reflect personal environmental exposure in a dose-dependent manner or by differential exposure status. In the current study, we selected three gene variants (rs1229984 (*ADH1B*), rs1260326 (*GCKR*), and rs13107325 (*SLC39A8*) that were significantly associated with alcohol consumption based on the results of the previous two GWAS studies of genetic determinants of alcohol consumption [23,24]. When participants in the population are grouped by particular genotypes that associate with differences in alcohol consumption, they should be similar in all respects, except alcohol consumption, with one group consisting mainly of never-drinkers or light drinkers and the other primarily including moderate to heavy drinkers [21,22,23,24]. In this way, MR makes it possible to randomly group participants.

The UK Biobank, a large population-based prospective cohort, recruited more than 500,000 participants aged 40–69 in 2006–2010 across the United Kingdom [25]. Detailed information on alcohol consumption, other lifestyle factors, and blood, saliva, and urine samples were rigorously collected from all participants in 2006–2010 [25]. In addition, the UK Biobank began releasing COVID-19 test results from March 16, 2020. Date on ICU (Intensive care unit) admissions and deaths of positive COVID-19 patients were also released in a timely manner. In order to better understand the potential impact of alcohol consumption on the risk of SARS-CoV-2 infection and the progression of COVID-19, we applied the Mendelian randomization approach [26] to evaluate the causal association among participants enrolled in the UK Biobank.

## 2. Materials and Methods

This study is consistent with the Strengthening the Reporting of Observational Studies in Epidemiology (STROBE) guideline. The study did not have a pre-registered or published analysis plan. UK Biobank has obtained Research Tissue Bank (RTB) approval from its ethics committee and this study was also approved by the Institutional Review Boards of the Cleveland Clinic (IRB number: 19582).

### 2.1. Study Population from UK Biobank

A total of 13,502 participants in the UK Biobank were tested for COVID-19 between 16 March and 27 July 2020. We excluded participants without alcohol consumption data (*n* = 67) and those without genotype data (*n* = 515). Finally, 12,937 participants were included in our study. Participants enrolled in UK Biobank have signed consent forms.

### 2.2. Exposure of Interest

The primary exposure of interest was alcohol consumption. Alcohol consumption data on participants enrolled by the UK Biobank obtained through a self-completed touchscreen questionnaire at the time of enrollment. Participants were asked about their current drinking status (never, previous, current). For current drinkers, they were then asked about the frequency of intake and their average weekly and monthly consumption (the unit is a standard drink) of a range of beverage types (fortified wine, spirits, beer plus cider, red wine, champagne plus white wine). 

According to the NIAAA’s classification criteria [27], we classified participants as heavy drinkers (>7 drinks per week for women; >14 drinks per week for men), moderate drinkers (4–7 drinks per week for women; 4–14 drinks per week for men), light drinkers (3 drinks or fewer per week), and never or infrequent drinkers (special occasions only or 1–3 times a month). We also classified heavy drinkers, moderate drinkers, and light drinkers as frequent drinkers and those who never drank or drank infrequently as non/infrequent-drinkers.

### 2.3. Genetic Data 

UK Biobank released genetic sequence data from 488,377 individuals genotyped for 847,441 genetic variants in July 2017. Participants were genotyped on either the UK BiLEVE genotyping array (*n* = 49,950; 807,411 markers) or the UKB Axiom Array (*n* = 438,427; 825,927 markers). After filtering for genetic variants available on both genotyping arrays and sample quality control processes [25], 488,377 participants with 805,426 single nucleotide variants were available in the release. Detailed information about genotyping and imputation in the UK Biobank has been described previously [25]. 

Previous genome-wide association study (GWAS) of alcohol consumption in 941,280 individuals of European descent [24] identified 7 independent genome-wide significant nonsynonymous variants: rs1229984 (*ADH1B*), rs28929474 (*SERPINA1*), rs11692465 (*ACTR1B*), rs1260326 (*GCKR*), rs13107325 (*SLC39A8*), rs3803800 (*TNFSF12–13*), and rs3748034 (*HGFAC*), which were significantly associated with alcohol consumption. In addition, a prior GWAS [23] using data from UK Biobank and Genetic Epidemiology Research in Adult Health and Aging (*GERA*) datasets identified 6 SNPs (rs1229984 (*ADH1B*); rs13130794 (*KLB*); rs144198753 (*BTF3P13*); rs1260326 (*GCKR*); rs13107325 (*SLC39A8*); rs11214609 (*DRD2*)) significantly associated with high alcohol consumption. 

Although the variant rs1229984 has been successfully used as an instrumental variable in the causal estimation of alcohol consumption and assortative mating [28] and chronic widespread pain in the participants of UK biobank [29], the use of this single SNP may not adequately explain genetic variance in alcohol consumption because of the low minor allele frequency (2.2%) of rs1229984 in the UK Biobank [28]. In order to overcome the potential for weak instrument bias, we generated an allele score utilizing three hub SNPs: rs1229984 (*ADH1B*), rs1260326 (*GCKR*), and rs13107325 (*SLC39A8*), based on the results of the previous two GWAS [23,24]. These three SNPs were directly genotyped on both the UK BiLEVE and UKB Axiom Arrays and the missingness of these SNPs in participants selected in this study was less than 1%. Other SNPs were excluded because they were not directly genotyped (805,426 markers) on the UK Biobank arrays. Detailed information about the three selected SNPs included in this study is shown in Table S1**.** The allele score was calculated per individual as the weighted or unweighted sum of the number of fast alcohol metabolizing alleles of each SNP, whereas the effect of each SNP on alcohol consumption provided in the GERA database (Table S1) was used as weight for the calculation of the weighted allele score [23,30].

### 2.4. Other Potential Confounding Risk Factors for COVID-19

A number of potential risk factors for COVID-19 were obtained through the touchscreen questionnaire at the time of enrollment in 2006–2010: inpatient hospital, death register, and genotype data: age at time of COVID-19 test, sex, race (classed as white and non-white ethnic background), body mass index (BMI), blood type, smoking status (no, only occasionally, most or all days), comorbidities (alcohol related diseases, upper gastrointestinal diseases, chronic lower respiratory diseases, chronic heart diseases, diabetes mellitus, dementia, liver cirrhosis and/or liver failure, renal failure, tumor, and acquired immunodeficiency syndrome (AIDS)). In addition, at the time of enrollment in 2006–2010, trained nurse measured weight and standing height of participants. BMI was calculated by dividing weight (kg) by the square of standing height (m^2^). BMI was categorized into four groups according to the WHO classification [31]: underweight (<18.5 kg/m^2^), normal weight (18.5–24.9 kg/m^2^), overweight (25–29.9 kg/m^2^), and obesity (≥30 kg/m^2^).

ICD-10 codes (International Classification of Diseases, Tenth Revision) were used to identify comorbidities and the cause of death from medical records and death records.

Alcohol use disorder, alcohol liver diseases, alcohol pancreatitis, alcoholic gastritis, alcoholic cardiomyopathy, alcoholic psychosis, alcoholic myopathy, alcoholic polyneuropathy, and degeneration of the nervous system due to alcohol were uniformly classified as alcohol related diseases. Upper gastrointestinal disease events were defined as participants with gastroesophageal reflux disease (GERD), esophagitis, gastritis/duodenitis, or peptic ulcer. Chronic obstructive pulmonary disease (COPD), asthma, emphysema, and bronchitis/bronchiectasis were uniformly classified as chronic lower respiratory disease events. Chronic cardiac events were defined as participants with hypertensive, chronic ischemic heart disease, or heart failure. All disease-related ICD 10 codes are shown in the Table S2**.**

### 2.5. Ascertainment of Outcomes 

The primary outcome was rate of positive SARS-CoV-2 tests, the secondary outcome was the mortality in COVID-19 positive patients. Data on SARS-CoV-2 test results provided by the UK Biobank covered England, but not Scotland and Wales. Follow-up for mortality was conducted to 27 June 2020 through linkage from National Death Registries. Deaths registered in UK Biobank with diagnosis U071 as primary or secondary cause were considered COVID-19 related deaths.

In this study, we selected participants from England in the database and defined the occurrence of outcomes as 0 = non-occurrence, 1 = occurrence. COVID-19 death event (*n* = 287) was collected through latest death record with ICD10 code U071 and other deceased COVID-19 positive patients without the corresponding code.

### 2.6. Statistical Analysis

All analyses were performed using Stata (Version 14.0; Stata Corp, College Station, TX, USA). Weekly alcohol consumption was natural log-transformed to meet parametric assumptions. Categorical variables were tested for association using Chi-squared test or Fisher’s exact test if more than 20% of cells had expected frequencies <5. For multiple comparisons correction, the false discovery rate (FDR) was calculated using the Benjamini-Hochberg method [32] and an adjusted *p* value (*q*-value) < 0.10 were considered statistically significant.

Alcohol consumption was classified in three ways: (1) A four-level exposure categorical variable including never or infrequent drinkers, light drinkers, moderate drinkers, and heavy drinkers; (2) a binary exposure variable comparing non/infrequent-drinkers to frequent drinkers; and (3) a log-transformed continuous variable of weekly self-reported alcohol consumption among frequent drinkers. 

To reduce the potential confounding effects of factors other than alcohol intake on outcomes, propensity score matching (PSM) [33] was applied to match non/infrequent-drinkers and frequent drinkers without replacement when we evaluated the associations between alcohol consumption and outcomes. We included variables [34] previously reported to be associated with a higher risk of COVID-19 as the matching factors for PSM. Factors including age, sex, BMI categories, current smoking status, alcohol related diseases, asthma, emphysema, COPD, bronchitis/bronchiectasis, esophagitis, gastritis/duodenitis, peptic ulcer, GERD, hypertensive, chronic ischemic heart disease, heart failure, diabetes, dementia, renal failure, liver cirrhosis and/or liver failure, tumor, and AIDS.

We applied two approaches to evaluate the relationship of alcohol consumption with outcomes: logistic or Cox regression association analysis and Mendelian randomization analysis [26]. The logistic regression was applied to evaluate the relationship between alcohol intake and the odds of SARS-CoV-2 infection. The Cox regression analysis was applied to evaluate the relationship between alcohol consumption and the risk of death in COVID-19 positive patients.

We used Mendelian randomization based on three assumptions: (1) the instrumental variable (weighted or unweighted allele score) would not be associated with potentially confounding factors of the risk of COVID-19; (2) the instrumental variable was significantly associated with exposure factor-alcohol consumption; (3) the instrumental variable was only associated with the outcomes through the exposure of interest. We evaluated these three assumptions using linear or logistic regression.

For the Mendelian randomization analysis, the two-stage residual inclusion (2SRI) method [26] was used to calculate the causal effect of alcohol consumption on the outcomes, using the weighted or unweighted allele score as the instrumental variables. The 2SRI method requires adjustment due to different classification methods and variable types (binary or continuous variable) of alcohol consumption and outcomes. For the association of alcohol intake with the risk of SARS-CoV-2 infection, we utilized the adjusted 2SRI method. In the first stage, the association between alcohol intake and the instrumental variable in all participants who had been tested for COVID-19 was evaluated using a logistic regression model (binary exposure of non/infrequent-drinkers and frequent drinkers) or a linear regression (continuous variable of weekly alcohol consumption in frequent drinkers). In the second stage, the outcome was fit using residuals from the first stage in a logistic regression model. For the risk of death in COVID-19 positive patients, the residuals from the first stage of all COVID-19 positive patients were included as covariates in a Cox regression model. Since the 2SRI method was not suitable for evaluating the association between the four-level categorical variable of alcohol consumption and outcomes, we only applied the logistic or Cox regression analysis for these associations. In addition, we found obesity and race were associated with both the selected SNPs and the risk of COVID-19 (all *q*-value < 0.10). These analyses were also conducted separately for participants with and without obesity, and in those of self-reported white ethnicity.

### 2.7. Sensitivity Analysis 

We performed three sensitivity analyses to evaluate the impact of specific subgroup of participants and different definitions of outcomes, respectively, on our analysis. First, in order to clarify the relationship between alcohol intake and outcomes in patients with overweight, but not patients with obesity, we selected participants with overweight for the sensitivity analysis. Second, because some COVID-19 positive patients were admitted to the ICU before they died or recovered and there was no data on life support for patients other than those in ICU, we considered ICU admission and death as serious clinical events and applied logistic regression and Mendelian randomization analyses to evaluate the association between alcohol intake and the risk of severe clinical outcomes. Finally, we conducted an additional comparative analysis between regular drinkers (heavy, moderate, and light drinkers) and never-drinkers after excluding infrequent drinkers, because we grouped never drinkers and infrequent drinkers together when comparing them with frequent drinkers. 

### 2.8. Mediation Analysis 

Obesity was considered as a potential intermediate factor because of its association with instrumental variables (rs1229984, rs1260326, and rs13107325 genotypes) of alcohol intake (Table 1) and the risk of COVID-19 [35]. The mediation analysis was applied to decompose the effect of alcohol on the risk of SARS-CoV-2 infection and the risk of death in COVID-19 patients into natural direct and indirect effects. The aim of the mediation analysis was to get the magnitude and the direction of the effect correct after properly handling the potential confounders. In this study, BMI was categorized into two groups as non-obese group = 0 and obese group = 1. Drinking status was also divided into two groups, frequent drinkers and non/infrequent-drinkers. The natural direct effect (NDE) was defined as the effect of alcohol consumption on the risk of COVID-19 that is not mediated by obesity. The natural indirect effect (NIE) was defined as the effect of alcohol consumption that is mediated through obesity. The product of direct and indirect effects was expressed as the total effect (TE). The mediation analysis was conducted using the med4way command in Stata, which is an appropriate method to perform mediation analysis with time-to-event outcomes.

## 3. Results

### 3.1. Characteristics of Participants

A total of 12,937 participants who had been tested for COVID-19 (from 16 March to 27 July 2020) were selected for our analysis according to the inclusion and exclusion criteria. Of these participants, 1570 (12.1%) tested positive for COVID-19 and 11,367 (87.9%) tested negative. Regarding the drinking status of these participants, 4486 (34.8%) were never or infrequently drinkers, 1156 (8.9%) were light drinkers, 3795 (29.3%) were moderate drinkers, and 3490 (27.0%) were heavy drinkers. As shown in Table S3, significant differences were observed in age, sex, race, blood type, current smoking, and comorbidities other than dementia, tumor, and AIDS between the non/infrequent-drinkers and frequent drinkers (all *q* < 0.10). Frequent drinkers tended to have a higher proportion of participants who were older than 65 years (72.0% vs. 69.3%), white (96.6% vs. 86.1%), and of normal weight (28.6% vs. 24.2%) compared to non/infrequent-drinkers. In contrast to the known deleterious health effects of heavy drinking [7,8,9,36], frequent drinkers had a lower rate of comorbidities (upper gastrointestinal diseases, asthma, heart failure, hypertensive, chronic ischaemic heart disease, diabetes mellitus, renal failure, and AIDS) than non/infrequent-drinkers (all *q* < 0.10). Since the participants in the UK Biobank are between the ages of 50 and 83, we hypothesized that there may be survivor bias between exposure factor (alcohol consumption) and multiple complications. Therefore, to reduce the potential for survivor bias and the effects of confounding factors on outcomes, the association between alcohol and risk of COVID-19 were evaluated in the PSM cohorts and the 2SRI method applied to perform causal inference on the effect of alcohol consumption and the outcomes [37]. 

### 3.2. Instrumental Variable Associations

Characteristics of participants by rs1229984, rs1260326, and rs13107325 genotypes are shown in Table 1. Since the number of participants with alleles rs1229984 and rs13107325, associated with rapid ethanol metabolism, was relatively small, we combined participants with one or two rapid metabolism alleles for comparison. Participants with one or two rapid metabolism alleles compared with those with two reference alleles tended to have a higher proportion of white participants and patients with obesity (all *q* < 0.10). No significant differences in age, sex, blood types, current smoking, or comorbidities were observed (all *q* > 0.10). 

**Figure 1:**
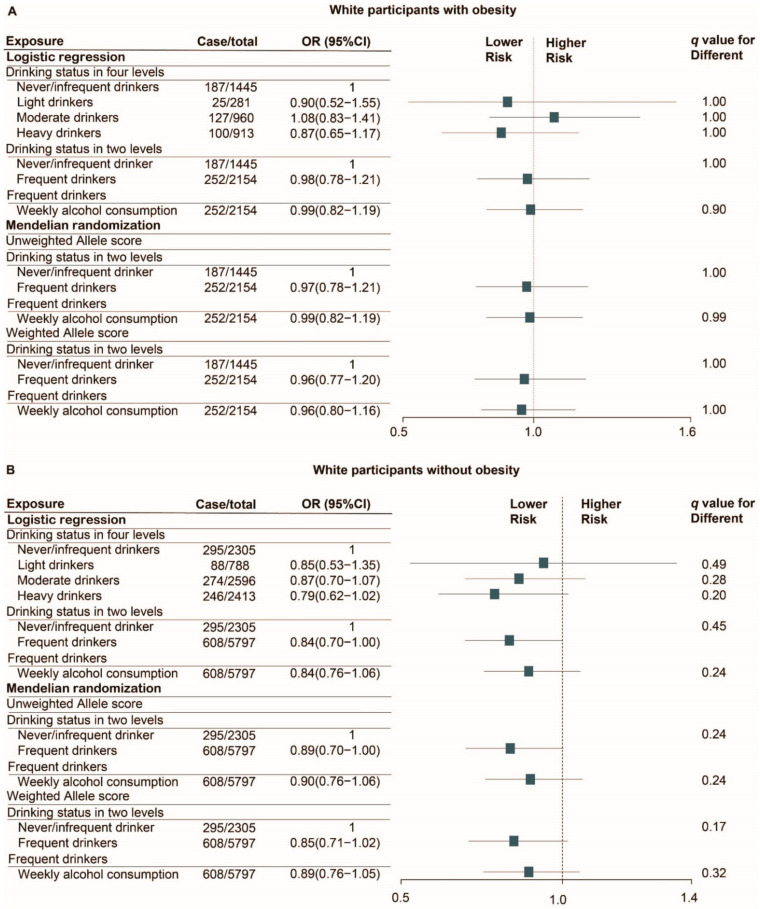
Logistic regression and Mendelian randomization analyses of the association of alcohol consumption with the risk of SARS-CoV-2 infection in white participants with (**A**) and without obesity (**B**). Analyses were performed in PSM cohort (Number of cases was counted from the cohort before PSM). Matching factors for PSM including age, sex, BMI categories, current smoking status, alcohol related diseases, asthma, emphysema, COPD, bronchitis/bronchiectasis, esophagitis, gastritis/duodenitis, peptic ulcer, GERD, hypertensive, chronic ischemic heart disease, heart failure, diabetes, dementia, renal failure, liver cirrhosis and/or liver failure, tumor, and AIDS. q-value was calculated by false discovery rate (FDR) method. Abbreviation: OR, odds ratio; HR, hazard ratio; CI, confidence interval; PSM, propensity score matching; BMI, body mass index; GERD, gastroesophageal reflux disease; COPD, chronic obstructive pulmonary disease; AIDS, acquired immunodeficiency syndrome.

White participants with one or two rapid metabolism alleles were less likely to be heavy-drinkers and on average drank less alcohol if they were frequent drinkers (Table 2 and Figure S1). These associations are consistent, but the difference between those with two or three more alleles is greater than those between zero and one (Figure 1). Associations of rs1229984, rs1260326, rs13107325 genotypes, and the weighted or unweighted allele score with alcohol consumption, were similar and significant (Table 2). The weighted allele score (F-test = 26.289) was more strongly related to alcohol consumption compared with other single SNPs and the unweighted allele score. Furthermore, we did not find an association between SNPs or allele scores and the odds of SARS-CoV-2 infection, the risk of severe clinical outcomes or death in patients with COVID-19 (Table S4).

### 3.3. Observational Associations and Instrumental Variable Associations

**Figure 2:**
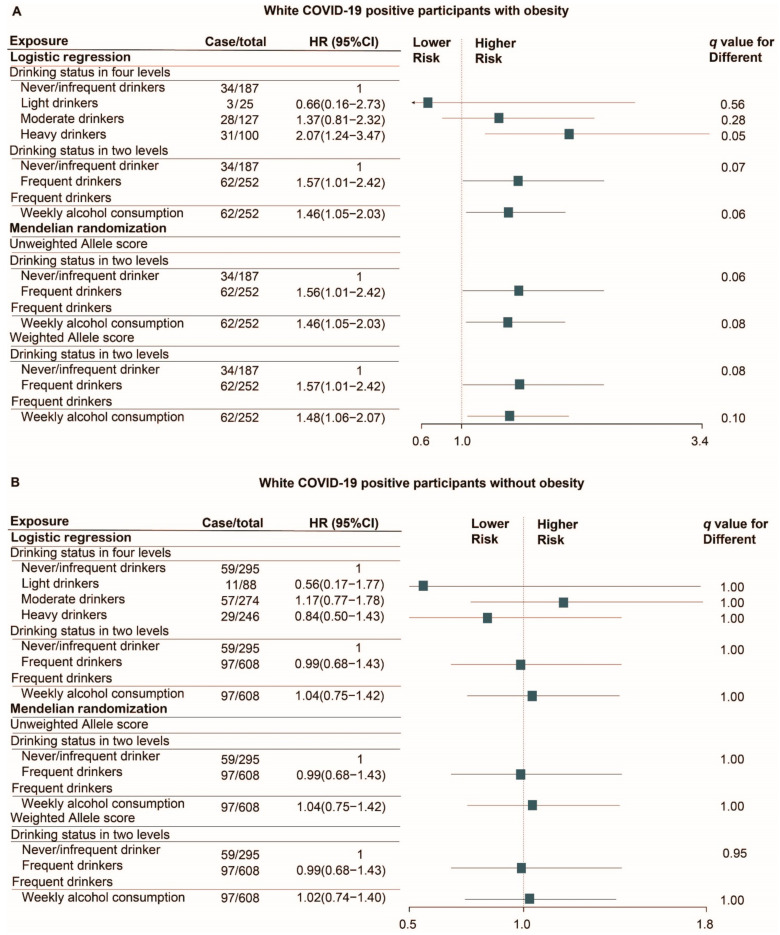
Cox regression and Mendelian randomization analyses of the association of alcohol consumption with the risk of death in white COVID-19 positive patients with (**A**) and without obesity (**B**). Analyses were performed in PSM cohort (Number of cases was counted from the cohort before PSM). Matching factors for PSM including age, sex, BMI categories, current smoking status, alcohol related diseases, asthma, emphysema, COPD, bronchitis/bronchiectasis, esophagitis, gastritis/duodenitis, peptic ulcer, GERD, hypertensive, chronic ischemic heart disease, heart failure, diabetes, dementia, renal failure, liver cirrhosis and/or liver failure, tumor, and AIDS. q-value was calculated by false discovery rate (FDR) method. Abbreviation: OR, odds ratio; HR, hazard ratio; CI, confidence interval; PSM, propensity score matching; BMI, body mass index; GERD, gastroesophageal reflux disease; COPD, chronic obstructive pulmonary disease; AIDS, acquired immunodeficiency syndrome.

The SARS-CoV-2 test positivity rate in white participants was 11.4% (1368/11,982) and the mortality rate of white patients with COVID-19 was 18.9% (258/1368). The ICU admission rate of white patients with COVID-19 was 8.3% (114/1368) and the average length of ICU stay was 9.5 days. After 1:1 PSM, 3750 non/infrequent-drinkers and 3750 matched frequent drinkers who were tested for SARS-CoV-2 were selected for evaluating the associations between alcohol consumption and the odds of SARS-CoV-2 infection. Among these participants, 482 non/infrequent-drinkers and 435 matched frequent drinkers who were tested positive for COVID-19 were selected for evaluating the association between alcohol consumption and the risk of worse clinical outcomes of COVID-19. The association of alcohol consumption with the odds of SARS-CoV-2 infection (Figure 1) and the risk of death in COVID-19 positive patients (Figure 2) are shown.

### 3.4. Alcohol and Risk of SARS-CoV-2 Infection

For the primary outcome, both logistic regression and Mendelian randomization analyses indicated that alcohol consumption within all three classifications of drinkers--the four-level categorical variable, the binary variable (non/infrequent-drinkers and frequent drinkers), and the continuous variable of weekly alcohol intake in frequent drinkers, was not associated with the risk of SARS-CoV-2 infection (All *q* > 0.10, Table S5). No association was detected in white participants either with or without obesity (All *q* > 0.10, Figure 1). In addition, there was no association between average weekly alcohol consumption and the risk of SARS-CoV-2 infection in either obese (OR = 1.07, 95%CI 0.92–1.24; *p* = 0.41) and non-obese (OR = 0.96, 95%CI 0.86–1.06; *p* = 0.38) cohort before PSM from Mendelian randomization analysis using the weighted allele score.

### 3.5. Alcohol and Risk of Death in White COVID-19 Positive Patients

The mortality rate of white patients with COVID-19 was 24.6% (62) among obese drinkers, 18.2% (34) among obese non/infrequent-drinkers, 16.0% (97) among non-obese drinkers, and 20.0% (59) among non-obese non/infrequent-drinkers. According to the results, we found that obese drinkers had higher rates of mortality than the other groups (*p* = 0.03).

**Figure 3:**
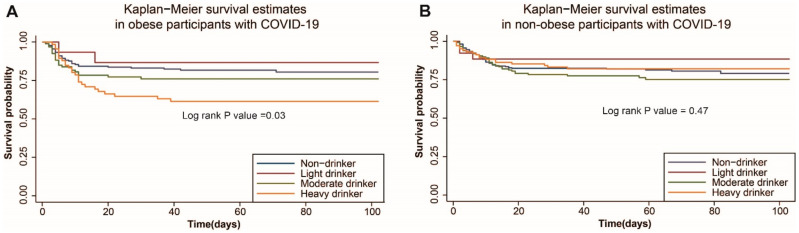
Survival probability of COVID-19 positive patient based on different drinking status in obese (**A**) and non-obese (**B**) groups.

For the risk of death in white COVID-19 positive patients, both Cox regression and Mendelian randomization analyses suggested that alcohol consumption within all three classifications of drinkers--the four-level categorical variable, the binary variable (non/infrequent-drinkers and frequent drinkers), and the continuous variable of weekly alcohol intake in frequent drinkers was not associated with the risk of death in white patients with COVID-19 (All *q* > 0.10, Table S5). However, in the subgroup analysis, white COVID-19 positive patients who were heavy drinkers with obesity had a higher risk of death (HR = 2.07, 95% CI 1.24–3.47; *q* = 0.05, Figure 2A). Both Cox regression (HR = 1.57, 95% CI 1.01–2.42; *q* = 0.07) and Mendelian randomization analyses using unweighted allele score (HR = 1.56, 95% CI 1.01–2.42; *q* = 0.06) or weighted allele score (HR = 1.57, 95% CI 1.01–2.42; *q* = 0.08) identified that white COVID-19 positive patients with obesity who reported consuming alcohol weekly were more likely to die compared with those drinking none or infrequently. In addition, we found higher alcohol consumption in frequent drinkers resulted in higher risk of death when analyzed by either Cox regression (HR = 1.46, 95% CI 1.05–2.03; *q* = 0.06) or Mendelian randomization analyses using the unweighted allele score (HR = 1.46, 95% CI 1.05–2.03; *q* = 0.08) or the weighted allele score (HR = 1.48, 95% CI 1.06–2.07; *q* = 0.10). However, these associations did not exist in non-obese patients with COVID-19 (*q* > 0.10, Figure 2B). Consistent results regarding the relationship between average weekly alcohol intake and the risk of death was found in both obese (HR = 1.42, 95% CI 1.05–1.91; *p* = 0.02) and non-obese (HR = 0.95, 95% CI 0.76–1.20; *p* = 0.68) cohorts before PSM from Mendelian randomization analysis using weighted allele score. As shown in Figure 3, Kaplan–Meier survival plots illustrated that heavy drinkers with obesity had a higher mortality than non/infrequent-drinkers (Log rank *p* value = 0.03), which was not observed in non-obese patients with COVID-19 (Log rank *p* value = 0.47).

### 3.6. Sensitivity Analysis

#### 3.6.1. Association between Alcohol Consumption and Outcomes in Overweight but Not Obese Patients

A total of 4869 overweight white participants were tested for SARS-CoV-2. The test positivity rate was 11.7% and the mortality rate of overweight patients with COVID-19 was 18.1% (103/568). Consistent with previous results for patients without obesity (Figure 1 and Figure 2), alcohol consumption was not associated with the risk of SARS-CoV-2 infection or death in overweight COVID-19 positive patients (*q* > 0.10, Table S6). 

#### 3.6.2. Risk of Severe Clinical Outcomes in White COVID-19 Positive Patients

The ICU admission rate of white patients with COVID-19 was 13.7% (34) among obese drinkers, 7.5% (14) among obese non-drinkers, 7.2% (44) among non-obese drinkers, and 7.1% (21) among non-obese non-drinkers. The average length of ICU stay was 9.3 days among obese drinkers, 10.6 days among obese non-drinkers, 9.0 days among non-obese drinkers, and 10.0 days among non-obese non-drinkers. According to the results, we found that obese drinkers had higher ICU admission rates than other groups (*p* = 0.02), but there was no statistically significant difference in the average length of ICU stay between the groups. 

**Figure 4:**
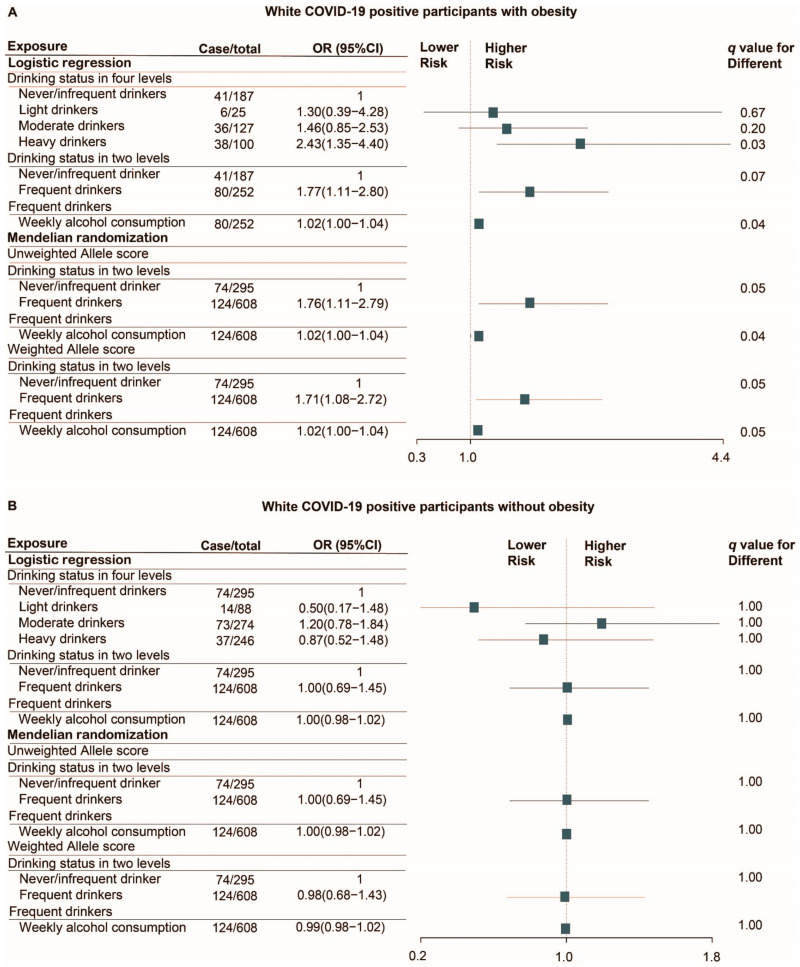
Logistic regression and Mendelian randomization analyses of the association of alcohol consumption with the risk of severe clinical outcomes in white COVID-19 positive patients with (**A**) and without obesity (**B**). Analyses were performed in PSM cohort (Number of cases was counted from the cohort before PSM). Matching factors for PSM including age, sex, BMI categories, current smoking status, alcohol related diseases, asthma, emphysema, COPD, bronchitis/bronchiectasis, esophagitis, gastritis/duodenitis, peptic ulcer, GERD, hypertensive, chronic ischemic heart disease, heart failure, diabetes, dementia, renal failure, liver cirrhosis and/or liver failure, tumor, and AIDS. q-value was calculated by false discovery rate (FDR) method. Abbreviation: OR, odds ratio; HR, hazard ratio; CI, confidence interval; PSM, propensity score matching; BMI, body mass index; GERD, gastroesophageal reflux disease; COPD, chronic obstructive pulmonary disease; AIDS, acquired immunodeficiency syndrome.

ICU admission and death in COVID-19 positive patients were grouped together as having severe clinical outcomes. The rate of severe clinical outcomes in white patients with COVID-19 was 31.7% (80) among obese drinkers, 21.9% (41) among obese non-drinkers, 20.4% (124) among non-obese drinkers, and 25.1% (74) among non-obese non-drinkers. According to the results, we found that obese drinkers had higher rates of mortality and serious clinical events than the other groups (*p* = 0.004). Since some of the patients were admitted to ICU before they were diagnosed with the SARS-CoV-2 infection, Cox regression model was not suitable for analyzing the association between alcohol consumption and severe clinical outcomes in the COVID-19 positive cohort without time-to-event data. For the risk of severe clinical outcomes in COVID-19 positive patients, both Cox regression and Mendelian randomization analyses failed to demonstrate a significant association between alcohol consumption and the risk of severe clinical outcomes in patients with COVID-19 (All *q* > 0.10). However, in the subgroup analysis, heavy drinkers with obesity had a higher likelihood of admission to ICU and death compared to non/infrequent-drinkers (OR = 2.43, 95% CI 1.35–4.40; *q* = 0.03, Figure 4A). Both logistic regression (OR = 1.77, 95% CI 1.11–2.80; *q* = 0.07) and Mendelian randomization analyses using unweighted allele score (OR = 1.76, 95% CI 1.11–2.79; *q* = 0.05) or weighted allele score (OR = 1.71, 95% CI 1.08–2.72; *q* = 0.05) identified that COVID-19 positive patients with obesity who reported consuming alcohol weekly were more likely to suffer severe clinical outcomes compared with those drinking none or infrequently. In addition, we found that the likelihood of serious clinical outcomes in frequent drinkers with obesity slightly increased with the average amount of alcohol consumed weekly based on the result of Mendelian randomization analysis using unweighted allele score (OR = 1.02, 95% CI 1.00–1.04, *q* = 0.10) and weighted allele score (OR = 1.02, 95% CI 1.00–1.04; *q* = 0.05, Figure 4A). However, these associations did not exist in non-obese patients with COVID-19 (*q* > 0.10, Figure 4B). 

#### 3.6.3. Differences in the Association between Alcohol Consumption and Outcomes between Frequent and Non-Drinkers

There was no association detected between alcohol consumption and the risk of SARS-CoV-2 infection in either patients with obesity (OR = 1.04, 95% CI 0.78–1.40; *q* = 1.00) or patients without obesity (OR = 1.01, 95% CI 0.79–1.29; *q* = 0.96, Table S7). Both Cox regression (HR = 5.05, 95% CI 1.74-14.67; *q* = 0.02) and Mendelian randomization analyses using weighted allele score (HR = 5.23, 95% CI 1.77–15.45; *q* = 0.04, Table S7) identified that COVID-19 positive patients with obesity who reported consuming alcohol weekly were more likely to suffer serious clinical outcomes compared with those who never drank alcohol. However, these associations did not exist in patients without obesity with COVID-19 (Table S7).

### 3.7. Mediation Analysis

We conducted a non-stratified mediation analysis to investigate the mediating effect of obesity on the relationship between alcohol consumption on risk of SARS-CoV-2 infection and serious clinical outcomes in patients with COVID-19. Assuming no interaction between the obesity and alcohol consumption, the risk of SARS-CoV-2 infection effect was not mediated by alcohol consumption (Estimate^NDE^ = −0.06, 95% CI: −0.20–0.08; *p* = 0.39, Table S8). The indirect effects of the obesity appeared null (Estimate^NIE^ = 0.0002, 95% CI: −0.003–0.002; *p* = 0.86), which indicated that obesity did not mediate the relationship between alcohol consumption and the risk of SARS-CoV-2 infection. In addition, for the risk of death in patients with COVID-19, the mediation effect of obesity was 0.009 (95% CI, −0.02–0.04; *p* = 0.57) with a negligible mediated proportion of 12.3% on the risk of serious clinical outcomes (Table S8).

## 4. Discussion

Using the UK Biobank cohort, we investigated whether alcohol consumption increased susceptibility to SARS-CoV-2 infection among 12,937 white participants who have been tested for SARS-COV-2, as well as whether there were worse outcomes among 1570 patients with COVID-19 using regression analyses and Mendelian randomization analysis.

In the Mendelian randomization study, we generated weighted and unweighted allele scores using three SNPs rs1229984 (*ADH1B*), rs1260326 (*GCKR*), and rs13107325 (*SLC39A8*) and applied the weighted and unweighted allele scores as the instrumental variables to assess the effect of alcohol consumption on outcomes. These three SNPs were randomly distributed among the white participants and were not associated with other tested confounders that may affect outcomes. Although rs1229984, rs1260326, and rs13107325 were significantly correlated with obesity, this issue was well addressed by our separate analysis based on whether the white participants were obese or not. In addition, the genetic variants used in the Mendelian randomization study were measured with precision. Thus, the Mendelian randomization approach more robustly handles measurement error and reverse causality compared to traditional observational approaches, and provides more reliable estimates of the potential underlying causal relationship of alcohol consumption with risk of COVID-19. 

Remarkably, both the traditional observation method and the Mendelian randomization method yielded consistent results indicating that alcohol consumption did not increase susceptibility to SARS-CoV-2 infection. However, frequent drinking, especially heavy drinking, was associated with worse outcomes of COVID-19 in patients with obesity, but not non-obese patients. Notably, the risk of worse clinical outcomes in frequent drinkers with obesity increased slightly with the average amount of alcohol consumed weekly. 

### 4.1. Possible Explanations for an Interaction between Alcohol Consumption and Worse Outcomes of COVID-19 in Patients with Obesity

According to our findings, frequent drinking was associated with poor outcomes of COVID-19 in patients with obesity, but not non-obese patients, suggesting potential interactions of obesity on the relationship between alcohol consumption and the severe COVID-19 outcomes. In addition, while mediation analysis failed to demonstrate a mediating effect of obesity on the relationship between alcohol consumption and the risk of COVID-19, there was a significant interaction between obesity and alcohol consumption on the risk of death in patients with COVID-19 (All *p* < 0.05, Table S9), consistent with our finding for the potential interactions between alcohol consumption and obesity in the development of severe COVID-19. Consistent with a previous report [35], our study found that patients with obesity were more susceptible to SARS-CoV-2 infection (OR = 1.26, 95% CI 1.09–1.45; *p* = 0.002) and had a higher risk of death (HR = 1.56, 95% CI = 1.12–2.17; *p* = 0.01) from COVID-19 in the whole cohort. The high expression of angiotensin-converting enzyme 2 receptor (ACE2) in adipocytes of patients with obesity may promote the entry of SARS CoV2 into host cells and turn adipose tissue into a potential target and reservoir of SARS CoV2 [38]. Moreover, adipose tissue, a major source of pro-inflammatory chemokines, cytokines, and adipokines, plays an important role in mediating inflammatory responses. Patients with obesity have higher concentrations of circulating tumor necrosis factor-alpha (TNF-a), interleukin-6, and C-reactive protein (CRP); this low grade chronic inflammatory state may be involved in initiating cytokine storms in patients with COVID-19 [35].

Alcohol consumption is associated with an increased risk of ARDS [11], which is a common manifestation of severe COVID-19. This is likely due to the dysregulation of both immune and non-immune host defense mechanisms in the airways, resulting in alveolar epithelial barrier dysfunction and alveolar macrophage immune dysregulation in response to heavy alcohol consumption [8,11]. However, the mechanisms for the potential interaction between alcohol and obesity in the progression of COVID-19 are not well understood. Previous work has found that chronic ethanol exposure in murine models impacts adipose tissue, phenocopying obesity in many aspects. Chronic ethanol feeding decreases glucose uptake by adipose tissue, increases immune cell infiltration and expression of inflammatory cytokines and adipokines [39]. Data from pre-clinical models also demonstrates that binge drinking and obesity synergistically induce steatohepatitis and fibrosis in the liver of mice via the induction of hepatic chemokines, inflammatory cytokines and neutrophil infiltration [40,41]. Serum ACE activity was found to be significantly higher in subjects with alcohol-use disorder compared with healthy controls, similar to the increases observed in patients with obesity [38,42]. In addition, among white COVID-19 positive patients, we found that frequent drinkers had a relatively lower proportion of obesity compared to non/infrequent drinkers (29.3% vs. 38.8%, *p* = 0.001), and obese drinkers have a higher mortality rate than obese non/infrequent-drinkers (24.6% vs. 18.2%, *p* = 0.02). Now the relationship between alcohol consumption and obesity is controversial [43], and many factors can affect alcohol to weight control, such as gender, age, type, amount, and frequency of alcohol consumed, sleeping habits, physical activity level, depression symptoms, chronic illness, medication use, psychosocial problems, etc. [43] Thus, although we speculated that the potential additive and/or synergistic effects of obesity and alcohol may lead to a significant deterioration of COVID-19 patients, the specific contributions of alcohol and obesity in the progression of COVID-19 still needs to be explored.

### 4.2. Strengths and Limitations

One of main advantages of this study is the application of Mendelian randomization to infer the causal relationships between alcohol consumption and the risk of SARS CoV2 infection and the severity of COVID-19 outcomes. To our knowledge, the associations between alcohol and risk of COVID-19 have not been previously explored using Mendelian randomization. Another major advantage of our study is the detailed data in a larger population-based prospective cohort including genotype data, alcohol consumption, and potential confounding risk factors. Almost all the confounding factors that have been reported to be associated with the risk of COVID-19 are available in the UK Biobank. Therefore, we were able to balance these factors in non/infrequent-drinkers and frequent drinkers using PSM to better assess the specific effect of alcohol consumption on the risk of COVID-19 in the traditional observational study and Mendelian randomization study. 

This study also has several limitations. First, since the majority of participants in the study were of British descent (93%) and only 195 non-white participants were infected with SARS CoV2, we could not investigate the impact of alcohol consumption on the risk of worse outcomes in non-white participants with COVID-19 using Mendelian randomization analysis. However, according to the results of the Chi-square test, we found heavy drinkers had a higher mortality than non-heavy drinkers (57.1% vs. 18.0%, *p* = 0.038) in the non-white group with obesity, which was not observed in non-obese group (20.0% vs. 8.0%, *p* = 0.220). Second, we are unable to assess the effect of changes in the alcohol consumption and BMI since subjects (*n* = 501,608) were enrolled in the UK Biobank in 2006-2010. Although the self-reported alcohol consumption data of some participants were updated in 2012 (*n* = 20,336), 2014 (*n* = 48,340) and 2019 (*n* = 3081), the number of these patients with COVID-19 was relatively small and the corresponding statistical analysis could not be performed. Only five COVID-19 positive patients had their alcohol consumption data updated in 2019 and there was no significant change in their drinking patterns (data not shown). Third, there was no association between alcohol consumption and risk of poor COVID-19 outcomes across the whole cohort or the non-obese cohort. However, there was a significant association in the obese cohort. Heuristically, the association may be thought to arise because patients with obesity are more likely to develop the adverse events under the burden of alcohol. In addition, previous research [44] has found participants tested for COVID-19 in the UK Biobank were highly selected for a range of behavioral, genetic, demographic, anthropometric, and cardiovascular traits when compared with the wider cohort. Due to the possibility of index-event bias and the challenge of interpreting observational results from such relatively unrepresentative samples from UK Biobank, caution should be used in interpreting our results until additional studies based on large databases are conducted to provide confirmatory evidence for our analysis. Finally, due to the limitations of the data in the UK Biobank, we could not assess the impact of alcohol consumption on the symptoms and some severe clinical outcomes (requirement of oxygen therapy, glucocorticoid therapy, and administration of invasive ventilation) of patients. To minimize this limitation, we classified ICU admission and death as the severe clinical outcomes of COVID-19 positive patients and found heavy drinkers had a higher risk of admission to ICU and death in patients with obesity compared to non/infrequent-drinkers.

## 5. Conclusions

Our study was the first to find that alcohol consumption, especially heavy drinking, is associated with a higher risk of suffering worse COVID-19 clinical outcomes in patients with obesity through both traditional regression analyses and Mendelian randomization analyses. In addition, alcohol consumption was not associated with either increased or decreased risk of SARS CoV2 infection. Our findings could help people understand the relationship between alcohol consumption and COVID-19, especially those who may drink excessively in the mistaken belief that alcohol consumption reduces the risk of SARS CoV2 infection [17,18]. However, due to the limitations of the UK Biobank data, we should treat the results with caution, as the evidence is not strong enough and more studies are needed to confirm our findings before they can be used for clinical guidance.

## Figures and Tables

**Table 1:** Characteristics of participants by rs1229984, rs1260326, and rs13107325 genotypes.

	ADH1B rs1229984			SLC39A8 rs13107325			GCKR rs1260326		
Variables	1/1 (Slow) *n* = 12,172	1/2 or 2/2 (Fast) *n* = 765	*q*-Value	1/1 (Slow) *n* = 11,160	1/2 or 2/2 (Fast) *n* = 1756	*q*-Value	1/1 (sLow) *n* = 4947	1/2 or 2/2 (Fast) *n* = 7920	*q*-Value
Age (years), *n* (%)			0.88			0.79			1.00
<65	3500 (28.8)	241 (31.5)		3274 (29.3)	463 (26.4)		1461 (29.5)	2252 (28.4)	
≥65	8672 (71.2)	524 (68.5)		7886 (70.7)	1293 (73.6)		3486 (70.5)	5668 (71.6)	
Male, *n* (%)	5977 (49.1)	358 (46.8)	0.81	5443 (48.4)	881 (50.2)	0.80	2452 (49.6)	3856 (48.7)	0.22
Race, *n* (%)			<0.001			<0.001			<0.001
No white	767 (6.3)	141 (18.5)		879 (7.9)	28 (1.6)		512 (10.4)	380 (4.8)	
White	11,359 (93.7)	623 (81.5)		10,252 (92.1)	1720 (98.4)		4416 (89.6)	7514 (95.2)	
BMI categories, *n* (%)									
Normal weight (18.5–24.9)	3201 (27.0)	221 (29.4)		3019 (27.7)	396 (23.0)		1263 (26.3)	2137 (27.6)	
Underweight (<18.5)	59 (0.5)	5 (0.7)	1.00	54 (0.5)	10 (0.6)	0.76	23 (0.5)	40 (0.5)	0.99
Overweight (25–29.9)	4891 (41.2)	341 (45.4)	1.00	4460 (41.0)	762 (44.3)	0.002	1946 (40.5)	3267 (42.2)	0.95
Obesity (≥30)	3719 (31.3)	184 (24.5)	0.06	3350 (30.8)	551 (32.1)	0.01	1577 (32.8)	2299 (29.7)	0.07
Blood type, *n* (%)									
OO	5128 (42.2)	330 (43.1)		4706 (42.2)	742 (42.4)		2098 (42.5)	3331 (42.1)	
AA + AO	416 (3.4)	31 (4.1)	0.91	388 (3.5)	58 (3.3)	0.97	169 (3.4)	274 (3.5)	0.91
BB + BO	1221 (10.0)	108 (14.1)	0.77	1155 (10.4)	172 (9.8)	0.85	520 (10.5)	800 (10.1)	0.53
AB	5390 (44.3)	296 (38.7)	0.93	4900 (44.0)	780 (44.5)	0.90	2154 (43.6)	3504 (44.3)	1.00
Current smoking, *n* (%)									
No	10,656 (87.6)	673 (88.0)		9775 (87.6)	1536 (87.6)		4318 (87.3)	6947 (87.8)	
Only occasionally	369 (3.0)	27 (3.5)	1.00	351 (3.1)	44 (2.5)	0.79	157 (3.2)	236 (3.0)	0.97
Most or all days	1137 (9.3)	65 (8.5)	0.87	1027 (9.2)	173 (9.9)	0.81	470 (9.5)	729 (9.2)	0.50
Comorbidities, *n* (%)									
Upper gastrointestinal diseases									
Oesophagitis	579 (4.8)	21 (2.7)	0.81	518 (4.6)	81 (4.6)	0.86	188 (3.8)	409 (5.2)	0.02
GERD	1363 (11.2)	74 (9.7)	0.99	1226 (11.0)	207 (11.8)	0.85	559 (11.3)	871 (11.0)	0.56
Peptic ulcer	428 (3.5)	25 (3.3)	1.00	396 (3.5)	57 (3.2)	0.56	175 (3.5)	277 (3.5)	1.00
Gastritis/duodenitis	1458 (12.0)	74 (9.7)	0.81	1311 (11.7)	218 (12.4)	0.58	570 (11.5)	953 (12.0)	0.99
Chronic lower respiratory diseases									
COPD	753 (6.2)	33 (4.3)	0.84	677 (6.1)	109 (6.2)	0.60	295 (6.0)	489 (6.2)	1.00
Emphysema	141 (1.2)	6 (0.8)	0.97	118 (1.1)	28 (1.6)	0.41	50 (1.0)	97 (1.2)	0.49
Bronchitis/Bronchiectasis	196 (1,6)	8 (1.0)	0.92	165 (1.5)	39 (2.2)	0.23	71 (1.4)	131 (1.7)	1.00
Asthma	1383 (11.4)	78 (10.2)	0.92	1259 (11.3)	199 (11.3)	0.97	538 (10.9)	914 (11.5)	0.46
Chronic heart diseases									
Heart failure	471 (3.9)	35 (4.6)	0.67	440 (3.9)	64 (3.6)	0.81	184 (3.7)	318 (4.0)	0.51
Hypertensive	4444 (36.5)	252 (32.9)	0.75	4065 (36.4)	625 (35.6)	0.53	1815 (36.7)	2857 (36.1)	1.00
Chronic ischaemic heart disease	1640 (13.5)	97 (12.7)	0.94	1496 (13.4)	239 (13.6)	0.93	628 (12.7)	1101 (13.9)	0.09
Diabetes mellitus	1475 (12.1)	91 (11.9)	0.94	1349 (12.1)	215 (12.2)	0.86	662 (13.4)	893 (11.3)	0.15
Serious liver diseases	88 (0.7)	3 (0.4)	0.89	79 (0.7)	12 (0.7)	0.90	29 (0.6)	62 (0.8)	0.47
Renal failure	897 (7.4)	52 (6.8)	0.93	807 (7.2)	139 (7.9)	0.72	388 (7.8)	556 (7.0)	0.52
Insomnia	9463 (77.8)	601 (78.6)	0.82	8675 (77.8)	1373 (78.2)	0.89	3887 (78.7)	6128 (77.4)	0.20
Dementia	91 (0.7)	2 (0.3)	0.88	80 (0.7)	13 (0.7)	0.93	44 (0.9)	49 (0.6)	0.16
Tumor	1246 (10.2)	77 (10.1)	0.90	1143 (10.2)	177 (10.1)	0.82	505 (10.2)	815 (10.3)	0.64
AIDS	11 (0.1)	2 (0.3)	0.72	12 (0.1)	1 (0.1)	0.83	4 (0.1)	8 (0.1)	1.00

*q*-value was calculated by false discovery rate (FDR) method. Abbreviation: BMI, body mass index; GERD, gastroesophageal reflux disease; COPD, chronic obstructive pulmonary disease; AIDS, acquired immunodeficiency syndrome.

**Table 2:** Association of genetic variations of ADH1B/SLC39A8/GCKR with alcohol consumption in white participants.

	Alcohol Consumption (Standard Drink/Weekly) in Frequent Drinkers (95% CI) (*n* = 8131)	OR of Being a Heavy Drinker in the Whole Cohort (95% CI) (*n* = 11982)
ADH1B one or two fast alleles vs. none	−2.78 (−4.08–−1.47)	0.525 (0.43–0.65)
F-test	17.42	—
*p*-value	<0.001	<0.001
SLC39A8 one or two fast alleles vs. none	−0.92 (−1.71–−0.13)	0.87 (0.78–0.98)
F-test	5.1	—
*p*-value	<0.001	0.022
GCKR one or two fast alleles vs. none	−0.79 (−1.36–−0.22)	0.92(0.84–0.99)
F-test	7.33	—
*p*-value	<0.001	0.039
Unweighted allele score	−0.73 (−1.07–−0.39)	0.89 (0.89–0.94)
F-test	17.47	—
*p*-value	<0.001	<0.001
Weighted allele score	−15.07 (−20.83–9.31)	0.05 (0.02–0.12)
F-test	26.29	—
*p*-value	<0.001	<0.001

F-test was calculated by linear regression analysis. Abbreviation: OR, odds ratio; CI, confidence interval; BMI, body mass index.

## Data Availability

This study used data from the UK Biobank (application number 59473). For details please contact access@ukbiobank.ac.uk. All other data are contained in the article and its supplementary information or available upon reasonable request.

## Supplementary Materials

The following are available online at https://www.mdpi.com/article/10.3390/nu13051592/s1, Table S1. Detailed information about the genetic variations of ADH1B/ SLC39A8/GCKR included in this study; Table S2. Disease diagnosis codes used by the UK Biobank; Table S3. Association of observed confounders with alcohol consumption; Table S4. Association of genetic variations of ADH1B/SLC39A8/GCKR with outcomes of interest in white participants; Table S5. Logistic/Cox regression and Mendelian randomization analyses of the associations of alcohol consumption with the risk of SARS-CoV-2 infection and the risk of death of COVID-19 in white participants; Table S6. Logistic/Cox regression and Mendelian randomization analyses of the associations of alcohol consumption with the risk of SARS-CoV-2 infection and the risk of death of COVID-19 in white participants who were overweight but not obese; Table S7. Logistic/Cox regression and Mendelian randomization analyses of the associations of alcohol consumption with the risk of SARS-CoV-2 infection and the risk of serious clinical outcomes of COVID-19 in white participants; Table S8. Mediation analysis of the effect of obesity on the associations of alcohol consumption with the risk of SARS-CoV-2 infection and the risk of death of COVID-19 in white participants; Table S9. Logistic/Cox regression analyses of the interaction effects between alcohol consumption and obesity in the risk of SARS-CoV-2 infection and the risk of death of COVID-19 in white participants; Figure S1. Association of combined ADH1B, SLC39A8, GCKR fast-allele score with average alcohol consumption levels (standard drink/weekly) in whole participants (A) and percentages of heavy-drinkers (B) in whole participants by unweighted allele score. The amount of alcohol consumed by non-drinkers was defined as zero.
