# Supplementary material for: Alcohol Consumption Is Associated with Poor Prognosis in Obese Patients with COVID-19: A Mendelian Randomization Study Using UK Biobank
Source: Nutrients. 2021 May 10;13(5):1592. doi: 10.3390/nu13051592 (PMC8152000; doi:10.3390/nu13051592)
Supplement: Supplementary file 1 [file nutrients-13-01592-s001.zip › nutrients-1196361-supplementary.pdf]

Table S1. Detailed information about the genetic variations of ADH1B/ SLC39A8/GCKR included in this study (Thompson et al., 2020).

SNP	Locus	Risk allele	Other allele	UK Biobank heavy alcohol drinker status (cases versus controls)			GERA database drinks/ week (among drinkers)	
				RAF	OR (95%CI)	P value	β(SE)	P value
**rs1229984**	ADH1B	C	T	0.98	1.58(1.48-1.70)	3.30×10^{-36}	0.19(0.02)	2.9×10^{-32}
**rs1260326**	GCKR	C	T	0.61	1.06(1.04-1.08)	2.60×10^{-8}	0.03 (0.03)	1.1×10^{-6}
**rs13107325**	SLC39A8	C	T	0.93	1.12(1.08-1.16)	1.60×10^{-8}	0.03(0.01)	0.02

Abbreviation: OR, odds ratio; CI, confidence interval; RAF, risk allele frequency; GERA, Genetic Epidemiology Research in Adult Health and Aging; ADH1B, alcohol dehydrogenase 1B; GCKR, glucokinase regulator; SLC39A8, solute carrier family 39 member 8.

Table S2. Disease diagnosis codes used by the UK Biobank

Diseases	Diagnosis code
**Alcohol related diseases**	
Alcohol use disorder	F10.0-F10.3
Alcohol liver diseases	K70.0-K70.9
Alcohol pancreatitis	K85.2 and K86.0
Alcoholic gastritis	K29.2
Alcoholic cardiomyopathy	I42.6
Alcoholic psychosis	F10.3-F10.9
Alcoholic myopathy	G72.1
Alcoholic polyneuropathy,	G62.1
Degeneration of the nervous system due to alcohol	G31.2
**Upper gastrointestinal diseases**	
Oesophagitis	K22.0-K22.9
GERD	K21.0, K21.9
Peptic ulcer	K25.0-K28.9
Gastritis/duodenitis	K29.0-K29.8
**Chronic lower respiratory diseases**	
COPD	J44.0, J44.1, J44.8, J44.9
Emphysema	J43.0-J43.2, J43.8, J43.9
Bronchitis/Bronchiectasis	J40, J41.0, J41.1, J42, J47
Asthma	J45.0, J45.1, J45.8, J45.9, J46
**Chronic heart diseases**	
Heart failure	I50.0-I50.9
Hypertensive	I10-I15.9
Chronic ischaemic heart disease	I23.0-I25.9
**Diabetes mellitus**	E10.0-E14.9
**Dementia**	F00.0-F03
**Liver cirrhosis and/or liver failure**	R18, K70.3, K71.7, K72.9, K74.3-K74.6, K76.6, K76.7, K70.4, K72, K72.0, K72.1, K72.9
**Renal failure**	N17.0-N19
**AIDS**	B20.0-B24

Abbreviation: GERD, gastroesophageal reflux disease; COPD, chronic obstructive pulmonary disease; AIDS, acquired immunodeficiency syndrome.

Table S3. Association of observed confounders with alcohol consumption

Variables	Non-drinkers (n=4496)	Frequent drinkers (n=8441)	Light drinkers (n=1156)	Moderate drinkers (n=3795)	Heavy drinkers (n=3490)	q-value
**Age (years), n(%)**						0.002
<65	1379(30.7)	2362(28.0)	330(28.5)	1012(26.7)	1020(29.2)	
≥65	3117(69.3)	6079(72.0)	826(71.5)	2783(73.3)	2470(70.8)	
**Male, n(%)**	1648(36.7)	4687(55.5)	414(35.8)	2502(65.9)	1771(50.7)	<0.001
**Race, n(%)**						<0.001
No white	622(13.9)	286(3.4)	56(4.9)	149(3.9)	81(2.3)	
White	3851(86.1)	8131(96.6)	1090(95.1)	3636(96.1)	3405(97.7)	
**BMI categories, n(%)**						<0.001
Normal weight (18.5-24.9)	1059(24.2)	2363(28.6)	365(32.2)	1008(27.2)	990(29.0)	
Underweight (<18.5)	26(0.6)	38(0.5)	12(1.1)	10(0.3)	16(0.5)	
Overweight (25-29.9)	1616(37.0)	3616(43.8)	459(40.5)	1691(45.6)	1466(43.0)	
Obesity (≥30)	1671(38.2)	2232(27.1)	296(26.1)	999(26.9)	937(27.5)	
**Blood type, n(%)**						0.002
OO	1853(41.3)	3605(42.8)	525(45.6)	1610(42.5)	1470(42.1)	0.002
AA+AO	181(4.0)	266(3.2)	45(3.9)	123(3.2)	98(2.8)	
BB+BO	514(11.5)	815(9.7)	108(9.4)	371(9.8)	336(9.6)	
AB	1941(43.2)	3745(44.4)	473(41.1)	1688(44.5)	1584(45.4)	
**Current smoking, n(%)**						<0.001
No	3908(87.0)	7421(88.0)	1064(92.1)	3445(90.8)	2912(83.5)	
Only occasionally	122(2.7)	274(3.2)	23(2.0)	104(2.7)	147(4.2)	
Most or all days	461(10.3)	741(8.8)	68(5.9)	244(6.4)	429(12.3)	
**Comorbidities, n(%)**						
**Upper gastrointestinal diseases**						

Oesophagitis	239(5.3)	361(4.3)	36(3.1)	175(4.6)	150(4.3)	0.01
GERD	601(13.4)	836(9.9)	121(10.5)	356(9.4)	359(10.3)	<0.001
Peptic ulcer	187(4.2)	266(3.2)	28(2.4)	117(3.1)	121(3.5)	0.01
Gastritis/duodenitis	664(14.8)	868(10.3)	126(10.9)	374(9.9)	369(10.5)	<0.001
**Chronic lower respiratory diseases**						
COPD	340(7.6)	446(5.3)	43(3.7)	193(5.1)	210(6.0)	<0.001
Emphysema	63(1.4)	84(1.0)	8(0.7)	44(1.2)	32(0.9)	0.11
Bronchitis/Bronchiectasis	78(1.7)	126(1.5)	25(2.2)	50(1.3)	51(1.5)	0.18
Asthma	640(14.2)	821(9.7)	115(9.9)	346(9.1)	360(10.3)	<0.001
**Chronic heart diseases**						
Heart failure	216(4.8)	290(3.4)	29(2.5)	139(3.7)	122(3.5)	0.001
Hypertensive	1839(40.9)	2857(33.8)	372(32.2)	1291(34.0)	1194(34.2)	<0.001
Chronic ischaemic heart disease	699(15.5)	1038(12.3)	121(10.5)	514(13.5)	403(11.5)	<0.001
**Diabetes mellitus**	796(17.7)	770(9.1)	117(10.1)	393(10.4)	260(7.4)	<0.001
**Liver cirrhosis and/or liver failure**	43(1.0)	48(0.6)	6(0.5)	15(0.4)	27(0.8)	0.02
**Renal failure**	408(9.1)	541(6.4)	78(6.7)	257(6.8)	206(5.9)	<0.001
**Insomnia**	3578(79.7)	6486(76.9)	885(77.0)	2831(74.6)	2770(79.4)	<0.001
**Dementia**	37(0.8)	56(0.7)	13(1.1)	22(0.6)	21(0.6)	0.18
**Tumor**	461(10.3)	862(10.2)	114(9.9)	391(10.3)	357(10.2)	1.00
**AIDS**	5(0.1)	8(0.1)	2(0.2)	5(0.1)	1(0.03)	1.00
**COVID-19 positivity, n(%)**	643(14.3)	927(11.0)	126(10.9)	433(11.4)	368(10.5)	<0.001
**COVID-19 positive patients, n(%)**						
**Death**	115(17.9)	172(18.6)	14(11.1)	90(20.8)	68(18.5)	0.43
**ICU admission and death**	147(22.9)	219(23.6)	21(16.7)	115(26.6)	83(22.6)	0.58

q-value was calculated by false discovery rate (FDR) method. **Abbreviation:** BMI, body mass index; GERD, gastroesophageal reflux disease; COPD, chronic obstructive pulmonary disease; AIDS, acquired immunodeficiency syndrome; COVID-19, coronavirus disease 2019; ICU, intensive care unit.

Table S4. Association of genetic variations of ADH1B/SLC39A8/GCKR with outcomes of interest in white participants

Instrumental variables	OR of SARS-CoV-2 infection	HR of death in COVID-19 positive patients	OR of ICU admission and death in COVID-19 positive patients
**ADH1B one or two fast alleles vs. none**	0.97(0.75-1.25)	0.72(0.37-1.39)	0.59(0.31-1.14)
**P-value**	0.78	0.32	0.12
**SLC39A8 one or two fast alleles vs. none**	0.98(0.84-1.16)	1.12(0.80-1.57)	1.16(0.82-1.65)
**P-value**	0.84	0.52	0.39
**GCKR one or two fast alleles vs. none**	0.99(0.88-1.12)	0.90(0.70-1.16)	0.84 (0.65-1.08)
**P-value**	0.92	0.41	0.18
**Unweighted allele score**	0.99(0.93-1.07)	0.97(0.83-1.12)	0.92(0.79-1.07)
**P-value**	0.92	0.66	0.27
**Weighted allele score**	0.96(0.31-2.9)7	0.32(0.02-4.48)	0.11(0.01-1.63)
**P-value**	0.94	0.40	0.11

Abbreviation: OR, odds ratio; HR, hazard ratio; CI, confidence interval; ICU, intensive care unit.

Table S5. Logistic/Cox regression and Mendelian randomization analyses of the associations of alcohol consumption with the risk of SARS-CoV-2 infection and the risk of death of COVID-19 in white participants

Exposure and outcomes	The risk of SARS-CoV-2 infection			The risk of death		
	Case/total	OR (95%CI)	q-value	Case/total	HR (95%CI)	q-value
**Logistic/Cox regression**						
**Drinking status in four levels**						
Never/infrequent drinkers	637/3750	1		189/637	1	
Light drinkers	53/326	0.95(0.70-1.29)	0.83	8/53	0.48(0.23-1.02)	0.17
Moderate drinkers	337/1968	1.01(0.87-1.17)	0.90	113/337	1.21(0.96-1.54)	0.16
Heavy drinkers	224/1456	0.89(0.75-1.050)	1	83/224	1.29(0.99-1.67)	0.12
**Drinking status in two levels**						
Never/infrequent drinker	637/3750	1	0.71	189/637	1	0.14
Frequent drinkers	614/3750	0.96(0.85-1.08)		204/614	1.18(0.96-1.44)	
**Frequent drinkers**						
Weekly alcohol consumption	614/3750	0.93(0.84-1.04)	0.62	204/614	1.18(0.99-1.41)	0.10
**Mendelian randomization**						
**Unweighted allele score**						
**Drinking status in two levels**						
Never/infrequent drinker	637/3750	1	0.84	189/637	1	0.12
Frequent drinkers	614/3750	0.96(0.85-1.08)		204/614	1.18(0.96-1.44)	
**Frequent drinkers**						
Weekly alcohol consumption	614/3750	0.99(0.99-1.00)	0.49	204/614	1.01(1.00-1.02)	0.23
**Weighted allele score**						
**Drinking status in two levels**						
Never/infrequent drinker	637/3750	1	0.67	189/637	1	0.11
Frequent drinkers	614/3750	0.96(0.85-1.09)		204/614	1.18(0.96-1.44)	

**Frequent drinkers**						
Weekly alcohol consumption	614/3750	0.99(0.99-1.00)	0.81	204/614	1.01(1.00-1.02)	0.41

Analyses were performed in PSM cohort. Matching factors for PSM including age, sex, BMI categories, current smoking status, alcohol related diseases, asthma, emphysema, COPD, bronchitis/bronchiectasis, esophagitis, gastritis/duodenitis, peptic ulcer, GERD, hypertensive, chronic ischemic heart disease, heart failure, diabetes, dementia, renal failure, liver cirrhosis and/or liver failure, tumor and AIDS.

q-value was calculated by false discovery rate (FDR) method.

Abbreviation: OR, odds ratio; HR, hazard ratio; CI, confidence interval; PSM, propensity score matching; BMI, body mass index; GERD, gastroesophageal reflux disease; COPD, chronic obstructive pulmonary disease; AIDS, acquired immunodeficiency syndrome.

Table S6. Logistic/Cox regression and Mendelian randomization analyses of the associations of alcohol consumption with the risk of SARS-CoV-2 infection and the risk of death of COVID-19 in white participants who were overweight but not obese.

Exposure and outcomes	The risk of SARS-CoV-2 infection (Overweight)			The risk of death (Overweight)		
	Case/total	OR (95%CI)	q-value	Case/total	HR (95%CI)	q-value
**Logistic and Cox regression**						
**Drinking status in four levels**						
Never/infrequent drinkers	181/1383	1		34/181	1	
Light drinkers	52/431	0.88(0.48-1.60)	0.76	8/52	0.35(0.05-2.54)	1.00
Moderate drinkers	193/1622	0.97(0.75-1.25)	0.80	43/193	1.20(0.72-2.01)	1.00
Heavy drinkers	142/1433	0.77(0.56-1.04)	0.83	18/142	0.96(0.51-1.83)	0.91
**Drinking status in two levels**						
Never/infrequent drinker	181/1383	1	0.49	34/181	1	1.00
Frequent drinkers	387/3486	0.88(0.71-1.10)		69/387	1.05(0.66-1.67)	
**Frequent drinkers**						
Weekly alcohol consumption	387/3486	0.91(0.74-1.11)	0.53	69/387	1.19(0.79-1.77)	1.00
**Mendelian randomization**						
**Unweighted allele score**						
**Drinking status in two levels**						
Never/infrequent drinker	181/1383	1	0.60	34/181	1	1.00
Frequent drinkers	387/3486	0.88(0.71-1.10)		69/387	1.05(0.66-1.67)	
**Frequent drinkers**						
Weekly alcohol consumption	387/3486	0.99(0.98-1.00)	0.62	69/387	1.01(0.96-1.03)	1.00
**Weighted allele score**						
**Drinking status in two levels**						
Never/infrequent drinker	181/1383	1	0.47	34/181	1	0.98
Frequent drinkers	387/3486	0.90(0.72-1.13)		69/387	1.04(0.67-1.65)	

**Frequent drinkers**						
Weekly alcohol consumption	387/3486	0.99(0.98-1.00)	0.57	69/387	1.01(0.99-1.02)	1.00

Analyses were performed in PSM cohort. Matching factors for PSM including age, sex, BMI categories, current smoking status, alcohol related diseases, asthma, emphysema, COPD, bronchitis/bronchiectasis, esophagitis, gastritis/duodenitis, peptic ulcer, GERD, hypertensive, chronic ischemic heart disease, heart failure, diabetes, dementia, renal failure, liver cirrhosis and/or liver failure, tumor and AIDS.

q-value was calculated by false discovery rate (FDR) method.

Abbreviation: OR, odds ratio; HR, hazard ratio; CI, confidence interval; PSM, propensity score matching; BMI, body mass index; GERD, gastroesophageal reflux disease; COPD, chronic obstructive pulmonary disease; AIDS, acquired immunodeficiency syndrome.

Table S7. Logistic/Cox regression and Mendelian randomization analyses of the associations of alcohol consumption with the risk of SARS-CoV-2 infection and the risk of serious clinical outcomes of COVID-19 in white participants

Exposure and outcomes	The risk of SARS-CoV-2 infection			The risk of serious clinical outcomes		
	Case/total	OR (95%CI)	q-value	Case/total	HR (95%CI)	q-value
**Patients without obesity**						
**Logistic/Cox regression**						
**Drinking status in four levels**						
Never drinkers	96/618			52/96	1	
Light drinkers	31/190	1.06(0.68-1.65)	1.00	12/31	0.73(0.26-2.08)	0.71
Moderate drinkers	194/1260	0.99(0.76-1.29)	1.00	90/194	0.94(0.49-1.80)	0.85
Heavy drinkers	135/855	1.02(0.77-1.36)	1.00	66/135	0.75(0.38-1.48)	0.71
**Drinking status in two levels**						
Never drinker	96/618	1	0.96	52/96	1	0.78
Frequent drinkers	360/2305	1.01(0.79-1.29)		168/360	0.84(0.46-1.53)	
**Frequent drinkers**						
Weekly alcohol consumption	360/2305	0.92(0.80-1.07)	1.00	168/360	0.92(0.66-1.28)	0.70
**Mendelian randomization**						
**Drinking status in two levels**						
Never drinker	96/618	1	1.00	52/96	1	
Frequent drinkers	360/2305	1.02(0.80-1.30)		168/360	0.87(0.47-1.59)	0.74
**Frequent drinkers**						
Weekly alcohol consumption	360/2305	0.99(0.99-1.01)	1.00	168/360	0.99(0.97-1.02)	0.79
**Patients with obesity**						
**Logistic/Cox regression**						
**Drinking status in four levels**						
Never drinkers	68/400	1		33/68	1	

Light drinkers	22/136	0.94(0.56-1.59)	1.00	10/22	5.63(1.52-20.83)	0.03
Moderate drinkers	143/708	1.24(0.90-1.70)	1.00	76/143	4.59(1.52-13.89)	0.03
Heavy drinkers	89/601	0.85(0.60-1.20)	1.00	50/89	5.68(1.81-17.87)	0.01
**Drinking status in two levels**						
Never drinker	68/400	1	1.00	33/68	1	0.02
Frequent drinkers	254/1445	1.04(0.78-1.4-)		136/254	5.05(1.74-14.67)	
**Frequent drinkers**						
Weekly alcohol consumption	254/1445	0.94(0.80-1.11)	1.00	136/254	1.04(1.01-1.06)	0.04
**Mendelian randomization**						
**Drinking status in two levels**						
Never drinker	68/400	1	1.00	33/68	1	0.04
Frequent drinkers	254/1445	1.03(0.77-1.39)		136/254	5.23(1.77-15.45)	
**Frequent drinkers**						
Weekly alcohol consumption	254/1445	0.99(0.99-1.00)	1.00	136/254	1.04(1.01-1.07)	0.04

Analyses were performed in PSM cohort. Matching factors for PSM including age, sex, BMI categories, current smoking status, alcohol related diseases, asthma, emphysema, COPD, bronchitis/bronchiectasis, esophagitis, gastritis/duodenitis, peptic ulcer, GERD, hypertensive, chronic ischemic heart disease, heart failure, diabetes, dementia, renal failure, liver cirrhosis and/or liver failure, tumor and AIDS.

q-value was calculated by false discovery rate (FDR) method.

Abbreviation: OR, odds ratio; HR, hazard ratio; CI, confidence interval; PSM, propensity score matching; BMI, body mass index; GERD, gastroesophageal reflux disease; COPD, chronic obstructive pulmonary disease; AIDS, acquired immunodeficiency syndrome.

Table S8. Mediation analysis of the effect of obesity on the associations of alcohol consumption with the risk of SARS-CoV-2 infection and the risk of death of COVID-19 in white participants

Effect	The risk of SARS-CoV-2 infection		The risk of serious clinical outcomes	
	Estimate (95% CI)	P-value	Estimate (95% CI)	P-value
Direct effect not through moderator (Obesity)	-0.06(-0.20-0.08)	0.39	-0.15(-0.36-0.06)	0.16
Indirect effect through moderator (Obesity)	0.0002(-0.003-0.002)	0.86	0.01(-0.02-0.04)	0.57
Total cause effect	-0.04(-0.15-0.08)	0.56	-0.17(-0.32-0.02)	0.12
Proportion of mediation	4.45%		12.3%	

Analyses were performed in PSM cohort. Matching factors for PSM including age, sex, BMI categories, current smoking status, alcohol related diseases, asthma, emphysema, COPD, bronchitis/bronchiectasis, esophagitis, gastritis/duodenitis, peptic ulcer, GERD, hypertensive, chronic ischemic heart disease, heart failure, diabetes, dementia, renal failure, liver cirrhosis and/or liver failure, tumor and AIDS.

Abbreviation: CI, confidence interval; PSM, propensity score matching; BMI, body mass index; GERD, gastroesophageal reflux disease; COPD, chronic obstructive pulmonary disease; AIDS, acquired immunodeficiency syndrome.

Test for Interaction using Logistic/Cox regression analyses

Method

According to our findings, frequent drinking was associated with poor outcomes of COVID-19 in patients with obesity, but not non-obese, patients, suggesting the potential interactions of obesity on the relationship between alcohol consumption and the severe COVID-19 outcomes. Therefore, regression analysis was used to test the interaction effects between alcohol consumption and obesity in the risk of SARS-CoV-2 infection and the risk of death of COVID-19 in white participants. In this study, BMI was categorized into two groups as non-obese group=0 and obese group=1. Drinking status was also divided into two groups, frequent drinkers and non/infrequent-drinkers. The logistic regression model was applied to analysis the potential interactions of obesity on the relationship between alcohol consumption and the risk of SARS-CoV-2 infection. For the risk of death in COVID-19 positive patients, the interaction effects were evaluated using the Cox regression model. Analyses were performed in PSM cohort. P value <.05 were considered statistically significant.

Result

Our study found that patients with obesity were more susceptible to SARS-CoV-2 infection (OR=1.017, 95%CI 1.006-1.029; P=.002, Table S9) and had a higher risk of death (HR=1.201, 95%CI=1.016-1.420; P=.032) from COVID-19 in white participants. Alcohol consumption was not associated with the risk of SARS-CoV-2 infection and the risk of death in participants with COVID-19 (All P >.05, Table S9). In addition, there was a significant interaction between obesity and alcohol consumption on the risk of death in patients with COVID-19 (HR=1.395, 95%CI=1.107-1.757; P=.005). No significant interaction between obesity and alcohol consumption on the risk of SARS-CoV-2 infection (All P >.05).

Table S9. Logistic/Cox regression analyses of the interaction effects between alcohol consumption and obesity in the risk of SARS-CoV-2 infection and the risk of death of COVID-19 in white participants

Exposure and outcomes	The risk of SARS-CoV-2 infection		The risk of death	
	**OR (95%CI)**	**P-value**	**HR (95%CI)**	**P-value**
**BMI categories**				
No-obese group	1		1	
obese group	1.02(1.01-1.03)	0.002	1.20(1.02-1.42)	0.03
**Drinking status**				
Never/infrequent drinker	1		1	
Frequent drinkers	0.96(0.85-1.08)	0.48	1.18(0.96-1.44)	0.11
**Interaction test**				
BMI categories*Drinking status	1.08(0.93-1.26)	0.32	1.40(1.11-1.76)	0.01

Analyses were performed in PSM cohort. Matching factors for PSM including age, sex, BMI categories, current smoking status, alcohol related diseases, asthma, emphysema, COPD, bronchitis/bronchiectasis, esophagitis, gastritis/duodenitis, peptic ulcer, GERD, hypertensive, chronic ischemic heart disease, heart failure, diabetes, dementia, renal failure, liver cirrhosis and/or liver failure, tumor and AIDS.

Abbreviation: CI, confidence interval; PSM, propensity score matching; BMI, body mass index; GERD, gastroesophageal reflux disease; COPD, chronic obstructive pulmonary disease; AIDS, acquired immunodeficiency syndrome.

Reference

THOMPSON, A., COOK, J., CHOQUET, H., JORGENSON, E., YIN, J., KINNUNEN, T., BARCLAY, J., MORRIS, A. P. & PIRMOHAMED, M. 2020. Functional validity, role, and implications of heavy alcohol consumption genetic loci. *Sci Adv,* 6**,** eaay5034.

Figure S1

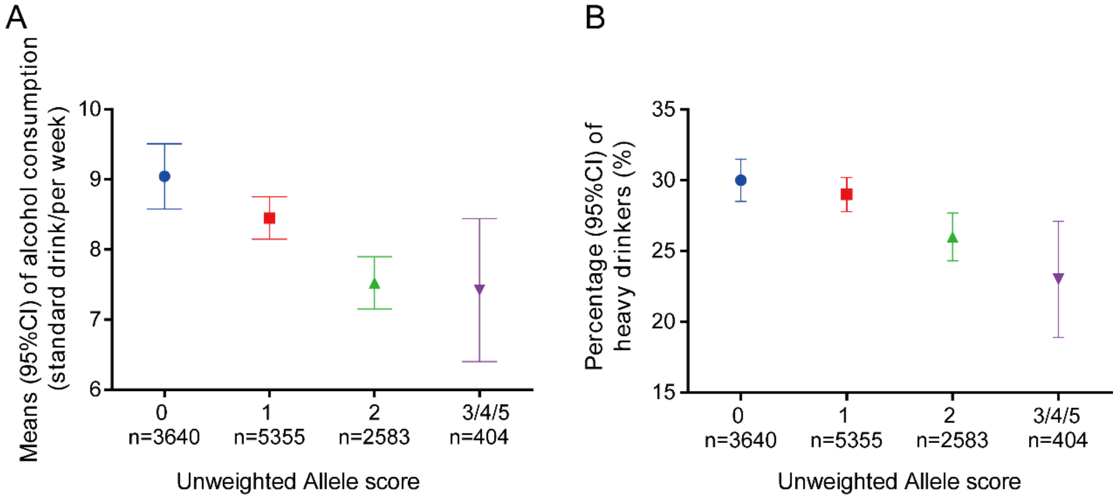

Figure S1. Association of combined ADH1B, SLC39A8, GCKR fast-allele score with average alcohol consumption levels (standard drink/weekly) in whole participants (A) and percentages of heavy-drinkers (B) in whole participants by unweighted allele score. The amount of alcohol consumed by non-drinkers was defined as zero.
